# Social media feedback and extreme opinion expression

**DOI:** 10.1371/journal.pone.0293805

**Published:** 2023-11-08

**Authors:** Elizaveta Konovalova, Gaël Le Mens, Nikolas Schöll

**Affiliations:** 1 Warwick Business School, University of Warwick, Coventry, United Kingdom; 2 Department of Economics and Business, Universitat Pompeu Fabra, Barcelona, Spain; 3 Barcelona School of Economics, Barcelona, Spain; 4 UPF-Barcelona School of Management, Barcelona, Spain; Universidade Federal de Ouro Preto, BRAZIL

## Abstract

On popular social media platforms such as Twitter, Facebook, Instagram, or Tiktok, the quantitative feedback received by content producers is asymmetric: counts of positive reactions such as ‘likes,’ or ‘retweets,’ are easily observed but similar counts of negative reactions are not directly available. We study how this design feature of social media platforms affects the expression of extreme opinions. Using simulations of a learning model, we compare two feedback environments that differ in terms of the availability of negative reaction counts. We find that expressed opinions are generally more extreme when negative reaction counts are not available than when they are. We rely on analyses of Twitter data and several online experiments to provide empirical support for key model assumptions and test model predictions. Our findings suggest that a simple design change might limit, under certain conditions, the expression of extreme opinions on social media.

## Introduction

When people create or share content on social media, they obtain quantitative information about the reactions of users in the form of numbers of ‘likes,’ ‘favourites,’ ‘thumbs up,’ or ‘re-tweets’ [1–3]. On Facebook and Instagram, such quantitative information consists in the number of likes given to a post. On Twitter, authors can see the number of comments, retweets, and likes received by their tweets. And on TikTok, users can see the number of likes, comments and shares received by their videos. Counts of such reactions are frequently seen as measures of popular success because they signal endorsement of the content [4–7]. Reaction counts are easily observed by content producers because they are prominently displayed with their posted content. By contrast, no count of negative reactions is available to content producers on these widely used platforms; their designs do not include one-button negative reactions.

In this paper, we conceive of counts of one-button reactions (e.g., ‘like’, ‘dislike’) to posted content as quantitative *feedback* received by content producers and study how the nature of the reaction counts provided to content producers affects the distribution of expressed opinions. We compare asymmetric feedback environments (only counts of positive reactions are provided to content producers) and symmetric feedback environments (both counts of positive and negative reactions are provided). Using analyses of a learning model in which content producers seek positive feedback, we show that, under a broad set of conditions, expressed opinions tend to be more extreme in asymmetric feedback environments than in symmetric feedback environments.

To understand the intuition for this result, consider the following stylized setting. Content producers on a social media platform can express their opinions about two candidate policies A and B to address a particular social issue by posting messages that strongly support A (AA), moderately support A (A), neither support A nor B (M), moderately support B (B), or strongly support B (BB). We consider two feedback environments that differ in terms of the reaction counts provided to content producers. In the asymmetric feedback environment (‘+’ environment), only positive reactions (or ‘no reaction’) are possible. In the symmetric feedback environment (‘+/−’ environment), positive and negative reactions are both possible. We find that, under a broad set of conditions, the proportion of expressed opinions that are extreme (AA or BB) is larger in the ‘+’ environment than in the ‘+/−’ environment.

Our argument relies on two sets of behavioural assumptions. The first set concerns the behaviour of content producers. First, content producers seek positive feedback. Recent experiments conducted by Facebook and LinkedIn support this assumption [8, 9]. They have found evidence that one of the main motivations for users to stay engaged on the platform is feedback. For example, researchers from LinkedIn experimented with changing the news feed order to increase visibility and feedback for users who had not yet decided if they wanted to continue posting on the platform [9]. By showing their posts more prominently in their friends’ news feeds, the platform ensured that content producers received more positive feedback to their posts and a higher share of them stayed engaged on the platform.

Second, we assume that content producers are more likely to repeat behaviour that leads to positive feedback. This ‘law-of-effect’ [10], ‘reinforcement learning’ [11] or ‘adaptive sampling’ assumption [12] is a reasonable choice heuristic when one tries to obtain positive payoffs [12–14]. Moreover, it is consistent with existing experimental evidence on sequential choice under uncertainty [14–17]. It is also consistent with studies of how social media users respond to feedback on Twitter [6] and Instagram [18].

The second set of assumptions concerns reactions to posted content. First, based on existing evidence that users of social media give more ‘likes’ to like-minded content [19], we assume that the probability a contact gives a positive reaction (‘+’) to an expressed opinion increases with its similarity to their own opinion. Second, we assume that the probability of a negative reaction (‘−’) decreases with similarity. We provide empirical support for both assumptions with online experiments and analyses of Twitter data (see also [1]).

Returning to our opinion expression example, these assumptions about reactions to posted content imply that, in the ‘+/−’ environment, moderate opinion expressions receive few ‘−’ because they are not extremely dissimilar to the opinion of any contact. But opinion expression that is extreme on one side of the opinion space (AA or BB) will receive ‘−’ from those with opposite opinions. Content producers who seek positive feedback, and react to the feedback they receive from their contacts in the form of counts of positive and negative reactions, will thus learn to express moderate opinions.

The dynamic of expressed opinions unfolds differently in the ‘+’ environment. Content producers learn about the number of their contacts who reacted positively to their expressed opinions but do not learn about the number of contacts who oppose their expressed opinion since they are not provided with a count of negative reactions. Most importantly, in the ‘+’ environment, the aggregated feedback received for expressing a moderate opinion is relatively less positive than in the ‘+/−’ feedback environment. Content producers who seek positive feedback will thus have a weaker tendency to express moderate opinions in the ‘+’ environment than in the ‘+’ environment. In other words, the availability of negative reaction counts moderates expressed opinions.

Our results contribute to previous theoretical work that noted the role of feedback in the extremization and polarization of opinion. Although existing work acknowledges feedback as a mechanism important for explaining patterns of opinion expression, it only considers symmetric feedback (i.e., both negative and positive feedback is possible and it has the same effect) [20, 21]. By comparing the symmetric and asymmetric settings, our analysis contributes to this literature by focusing on a design feature of social media platforms that has received scant attention. It emphasizes that this design feature has potentially important consequences for the propensity of users to express extreme opinions.

The mechanism on which we focus—feedback consisting of counts of positive (negative) reaction reactions—is different from the perspective advanced by work on ‘filter bubbles’ [22, 23] and the ‘backfire’ effect [24]. The filter bubble hypothesis—a commonly advanced explanation for political polarization that argues that social media users are mostly exposed to ideas consistent with their opinions—concentrates on the content of the messages read by users, and how the nature of the social network connections affects the information contained in these messages. Similarly, work on the backfire effect—a phenomenon according to which exposure to contrarian opinions leads to more entrenched views—assumes that people consume information in the form of messages posted on social media and links to news. We do not claim that work that focuses on the textual information people sample on social media and how they process it is unimportant. But our theoretical focus is on a different mechanism that could also contribute to opinion dynamics on social media. In this paper, we aim to analyze the implications of this single mechanism and leave it for further research to study how quantitative feedback and learning from information shared by other users interact in explaining opinion change and expression on social media.

In the following, we first describe a learning model designed to analyse the effect of the symmetric/asymmetric nature of the quantitative feedback environment on opinion expression and our main simulation results. Then we provide empirical evidence for key model assumptions using analyses of Twitter data and two experiments. Next, we report an experimental test of the main model predictions. Finally, we report additional simulation analyses that show how our main results change with modifications of the model assumptions.

## The effect of the feedback environment on the distribution of expressed opinions: Simulation analyses of a learning model

### Model description

A content producer posts a series of messages on a social media platform with the goal of generating positive reactions by their contacts. Each message lies at one of *K* discrete positions on the ideological spectrum. The position of the message posted at period *t* is denoted by *m*_*t*_ ∈ {1, …, *K*}. A fixed set of *J* contacts potentially react to the messages posted by the content producer. The contacts are uniformly distributed over positions on the ideological spectrum and respond to the posted message as a function of the distance between their positions and the message. After posting a message, the content producer observes the overall reaction of their contacts and updates their valuations of the position of the message they posted.

There are two feedback environments. In the symmetric environment (‘+/−’ environment), contacts who react to the message can react positively (‘+’) or negatively (‘−’), and content producers observe the counts of positive and negative reactions. In the asymmetric feedback environment (‘+’ environment), contacts cannot react negatively to the message, and the content producer observes the only count of positive (‘+’) reactions. In none of the environments does the content producer observe the count of contacts who did not react to their messages. The task environment is formally equivalent to a multi-armed bandit problem, which is one of the canonical settings for the analysis of how agents learn from experience while their goal is to maximize cumulative payoff. This model differs from standard models used to study polarization dynamics [25] in that some agents do not change their opinions (the ‘contacts’). This assumption was motivated by a concern for simplicity since a model in which all agents change their opinions would make it harder to understand the intuition for the key results. Moreover, the assumption that content producers aim to adapt to feedback but not their contacts maps well to the setting of politicians who adapt their discourse to fit the preferences of their constituents [6]. Our model thus does not speak to the conditions leading to the polarization of a full population, like [20]. But it casts light on the mechanisms leading to the polarization of content producers.

#### Choice rule

We implement the assumption that content producers seek positive feedback by assuming that the probability that the content producer posts a message at position *k* in period *t*, *ρ*_*k*,*t*_, increases with the content producer’s valuation of position *k*, *V*_*k*,*t*_:

$$\begin{array}{c} \rho_{k,t}=\frac{e^{cV_{k,t}}}{\sum_{i=1}^{K}e^{cV_{i,t}}}, \end{array}$$ (1)

where *c* > 0 is a parameter that characterizes the sensitivity of the choice probability to the valuations of the various positions. When *c* is large, the content producer is very likely to post a message at the position with the highest valuation. When *c* is close to 0, position choice is almost random.

#### Reactions by contacts

Contacts experience some ‘internal’ response to the message that might (or not) become an overt reaction (a click on a ‘one-button’ positive or negative reaction). The probability of a positive internal response is maximal if the message is at the same position on the ideological spectrum as the contact, and decreases with distance in the ideological spectrum between the position of the contact *o*_*j*_ ∈ {1, …, *K*} and the position of the message *m*_*t*_ ∈ {1, …, *K*}:

$$\begin{array}{c} l_{t}^{j}=l_{0}e^{-s_{l}\left|m_{t}-o_{j}\right|}, \end{array}$$ (2)

where *l*_0_ > 0 and *s*_*l*_ > 0.

The probability of a negative internal response is minimal if the message is at the same position as the contact, and increases with distance:

$$\begin{array}{c} d_{t}^{j}=d_{0}\frac{1-e^{s_{d}\left|m_{t}-o_{j}\right|}}{1-e^{s_{d}\left(K-1\right)}}, \end{array}$$ (3)

where *d*_0_ > 0 and *s*_*d*_ > 0. *l*_0_ (*d*_0_) characterizes the baseline tendency of giving a ‘+’ (‘−’) and *s*_*l*_ (*s*_*d*_) characterizes the sensitivity of the probability of a ‘+’ (‘−’) to the distance between the opinion of the contact and the position of the message.

In the ‘+’ environment, overt reactions can only be positive (‘+’) or absent. The probability that contact *j* gives a ‘+’ is $l_{t}^{j}$. In the ‘+/−’ environment, overt reactions can be positive (‘+’) or negative (‘−’) or absent. Contact *j* gives a ‘+’ with probability $l_{t}^{j}\left(1-d_{t}^{j}\right)$; they give a ‘−’ with probability $\left(1-l_{t}^{j}\right)d_{t}^{j}$.

These assumptions imply that in both environments, the probability of a positive reaction (‘+’) is decreasing with the distance between the message and the contact. In the ‘+/−’ environment, the probability of a negative reaction (‘−’) is increasing with distance.

#### Feedback perception by the content producers

We denote the feedback received by the message posted in period *t* as *F*_*t*_. The count of ‘+’ obtained by the message posted in period *t* is *L*_*t*_ and the count of ‘−’ is *D*_*t*_. In the ‘+/−’ environment, feedback is a linear combination of the two counts, consistent with research on averaging judgment models [26, 27]:

$$\begin{array}{c} F_{t}=\delta_{l}*L_{t}-\delta_{d}D_{t}, \end{array}$$ (4)

where *δ*_*l*_ and *δ*_*d*_ are weights of the counts of ‘+’ and ‘−’. In the ‘+’ environment, the weight of the count of negative reactions is set to zero: *δ*_*d*_ = 0.

#### Valuation updating

If the content producer posts a message at position *k* in period *t*, they update their valuation of that position based on the feedback *F*_*t*_. The new valuation is a weighted average of the previous valuation and the feedback:

$$\begin{array}{c} V_{k,t+1}=\left(1-b\right)V_{k,t}+bF_{t}, \end{array}$$ (5)

where *b* ∈ [0, 1] is the feedback weight. The valuation of position *k* does not change if the content producer does not post a message at this position in period *t*: *V*_*k*,*t*+1_ = *V*_*k*,*t*_.

This updating rule is frequently used to model how humans learn in sequential decision-making tasks and has received solid empirical support, both in psychology [28] and neuroscience [29]. Moreover, prior research has shown that the combination of a logistic choice rule (Eq 1) and this updating rule provides a good fit to experimental data on sequential choice under uncertainty [12].

### Main results

We simulated this learning model in the ‘+’ and ‘+/−’ environments. Unless otherwise noted, we use the following parameters: *K* = 5, *J* = 50, *c* = 0.3, *d*_0_ = *l*_0_ = 0.9, *s*_*l*_ = *s*_*d*_ = 1, *b* = 0.5, *δ*_*l*_ = *δ*_*d*_ = 0.5. We focus our analyses on the expression of extreme opinions (choice of position 1 or 5) versus the moderate opinion (choice of position 3).

In all simulations, the initial valuations of all the positions are the same. This implies that the position of the first message posted by a content producer follows a uniform distribution. This amounts to assuming that content producers do not have intrinsic preferences for a position on the ideological spectrum. This assumption is frequently made in models of politicians’ behaviour [6]. Valuation differences between positions are thus entirely driven by feedback.

**Fig 1:**
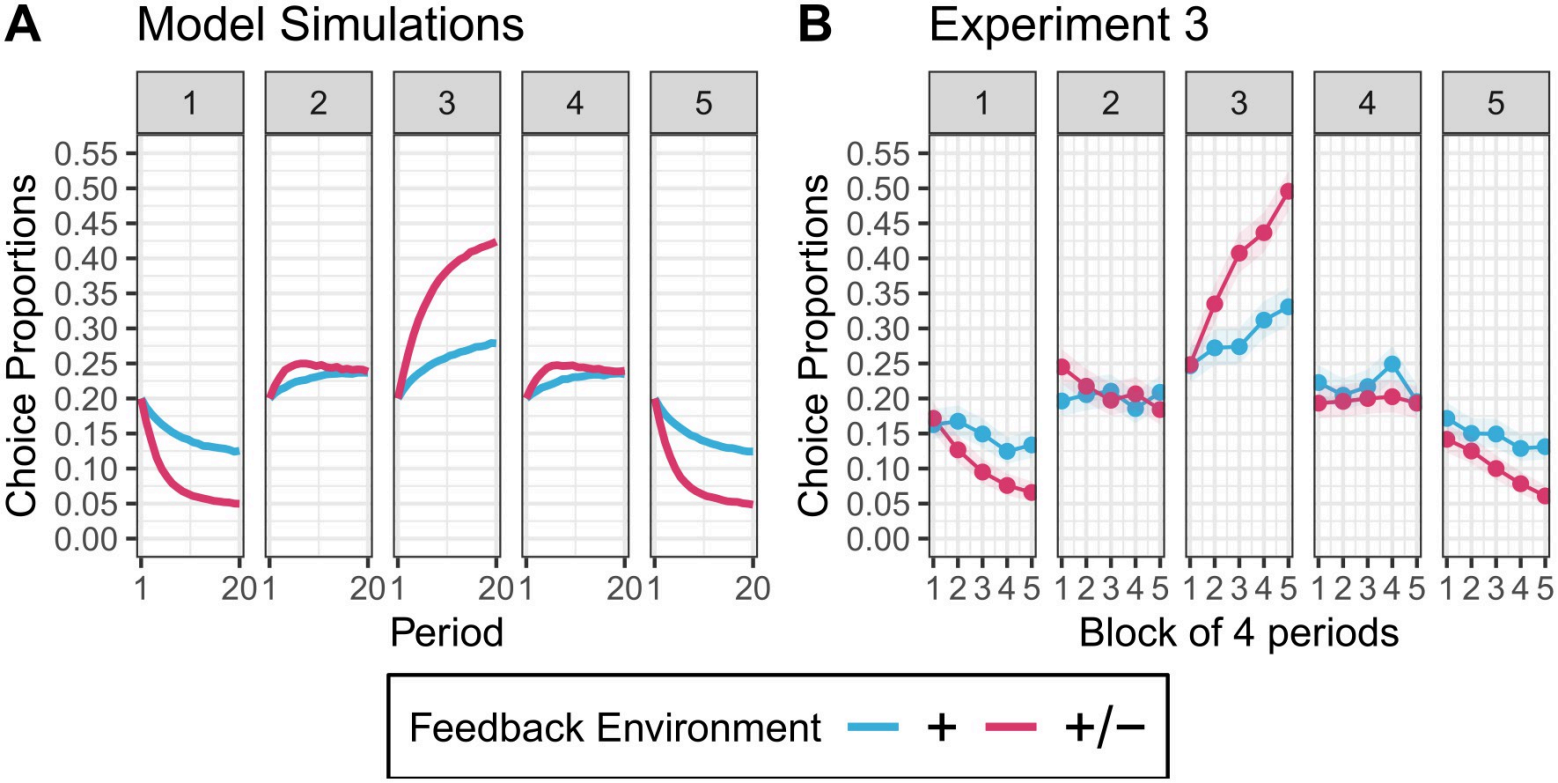
The effect of negative feedback on the expression of extreme opinion. Each panel refers to one of the *K* = 5 positions on the ideological spectrum (1 to 5). **A.** Simulation: Proportion of messages at each position in the two feedback environments. Simulations are based on 10,000 simulations with *J* = 50 contacts. **B.** Experiment 3: Choice proportions in the two feedback conditions. Each point is the proportion averaged over a block of 4 periods. The shaded ribbons represent 95% confidence intervals.

Panel A on Fig 1 shows the evolution of opinion expressions in the two feedback environments. The dynamics differ across environments. In the ‘+’ environment, the proportion of agents posting messages at one of the extreme positions (1 or 5) drops from 40% to 29% between periods 1 and 20. In the ‘+/−’ environment, this proportion decreases much more strongly: it drops to 10% in period 20. In summary, moving from the ‘+ environment’ to the ‘+/− environment’ leads to a reduction of extreme opinion expression (Panel B plots between environments differences).

Moving from the ‘+’ environment to the ‘+/−’ environment has a negative main effect on the feedback value *F*_*t*_, and thus the valuation of the *K* positions. But this absolute effect does not matter since the choice is driven by *differences* in the valuations of the positions (Eq 1). What is important is that the distributions of feedback over positions systematically differ between the two environments. Consider first the ‘+’ environment. Moderate positions are never very far from other positions (i.e., the distance from position 3 is never more than two), which implies that moderate messages can receive positive reactions from most contacts. In comparison, extreme positions are, *on average*, further away from contacts’ positions on the ideological spectrum. Extreme messages thus receive a lower count of positive reactions than moderate messages. Overall, this implies that starting with content producers who initially value all positions equally (and thus whose first message is uniformly distributed on the ideological spectrum), the distribution of expressed opinions becomes centred.

**Fig 2:**
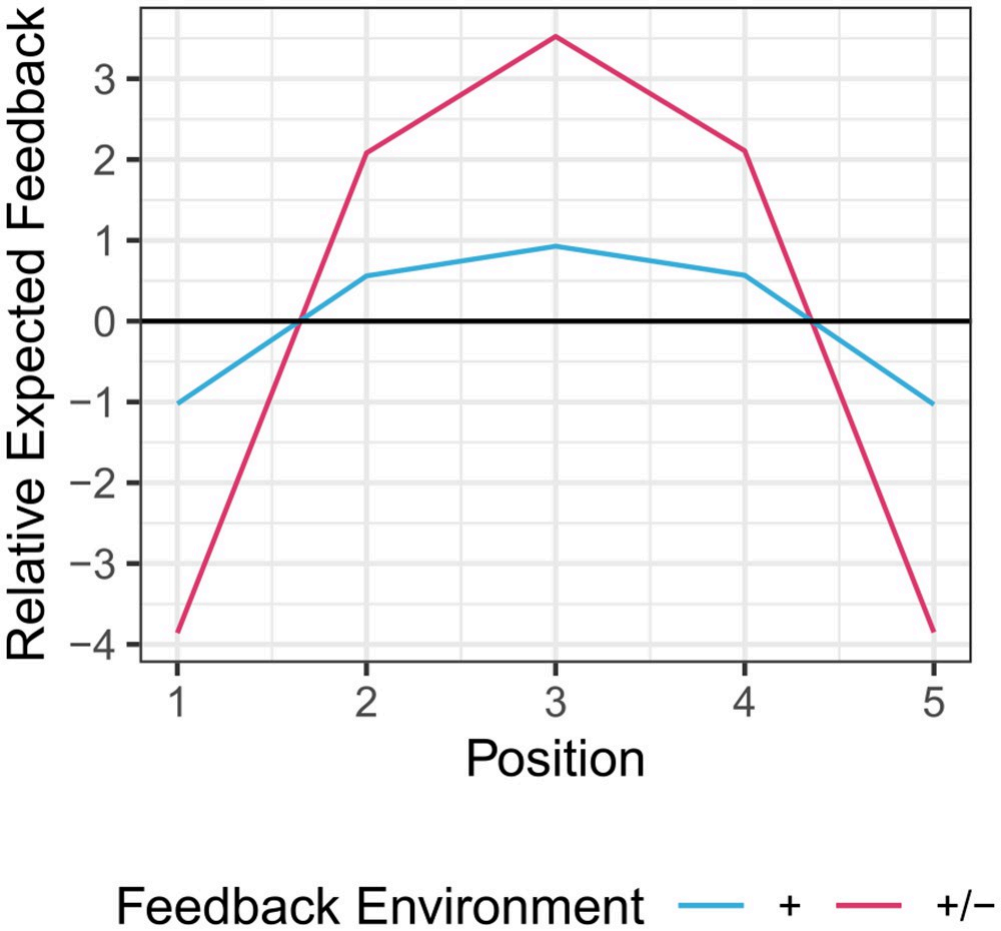
Average feedback relative to the within-environment mean feedback across positions. Feedback is relatively more positive at the central position and relatively more negative at the extreme positions.

In the ‘+/−’ environment, the distribution of positive reactions is almost the same as in the ‘+’ environment. But extreme messages receive much higher counts of negative reactions than moderate messages. This implies that the distribution of average feedback over positions is much more ‘peaked’ toward the centre of the ideological spectrum (see Fig 2). The moderating effect of feedback is thus stronger in the ‘+/−’ environment than in the ‘+’ environment. Ancillary simulations show that our main result, that opinion expression becomes less extreme in the ‘+/−’ environment than in the ‘+’ environment, is robust to the initial conditions chosen for the simulations (initial valuations of positions by content producers), after sufficiently many periods.

## Empirical evidence for model assumptions

In this section, we provide evidence for the main assumptions of our model using Twitter data and online experiments. First, we focus on the assumption regarding the reactions of contacts to the content. Specifically, using the tweeting behaviour of Spanish politicians, we show that the probability of a positive reaction is negatively associated with the distance between the content producer and the contact. Next, to remedy the fact that the Twitter results concern only positive reactions, we tested this assumption and the assumption that feedback givers are more likely to give negative feedback to a message when the opinion it expresses is far from theirs in an online study (Experiment 1). We chose the domain of commercial space travel because we anticipated that people would hold a variety of opinions about it. This topic was also used in Experiment 2, where we validated the model assumption about how people aggregate counts of positive and negative feedback.

All participants gave their informed written consent for inclusion before they participated in the studies. The study protocol was approved by the CIREP committee at Universitat Pompeu Fabra (Project #2018/7824/I). The tweets used in the analysis of Twitter data were downloaded in August 2022 via the Twitter API and analyzed in accordance with the developer policy and terms.

### Evidence from twitter

#### Methods

To provide empirical evidence in support of our assumption regarding how contacts select positive reactions to content (Eqs 2 and 3), we analyzed the retweeting behaviour of Spanish politicians. In the analysis, we use ‘retweets’ rather than ‘likes’ because Twitter data includes the identity of retweeters, but not the identity of those who give likes. We focus on reactions *by politicians* to tweets posted by other politicians because of issues related to the processing of personal data obtained from Twitter. European data protection regulations (GDPR) would not allow this kind of analysis with data of Twitter users who did not provide explicit consent or who are not public figures. Regular Twitter users do not expect that their data will be used to infer their positions on a political ideology space. Conversely, politicians cannot expect the political content of their public online discourse will not be analyzed.

Our data includes all tweets and retweets by a set of 633 politicians who were members of national or regional parliament during the 2016 to 2019 election cycle. To measure the ideological position of each tweet on the left-to-right spectrum based on the predicted categorization probabilities produced by a BERT-based text classifier [30, 31] and defined the position of a politician as the average position of the tweets they published—for another approach, see [1]. Finally, we defined the ideological distance between a tweet and a potential retweeter (the contact) as the absolute distance between the estimated ideological position of the tweet and the estimated ideological position of the contact (see Section S2 in the S1 File for details).

#### Results

**Fig 3:**
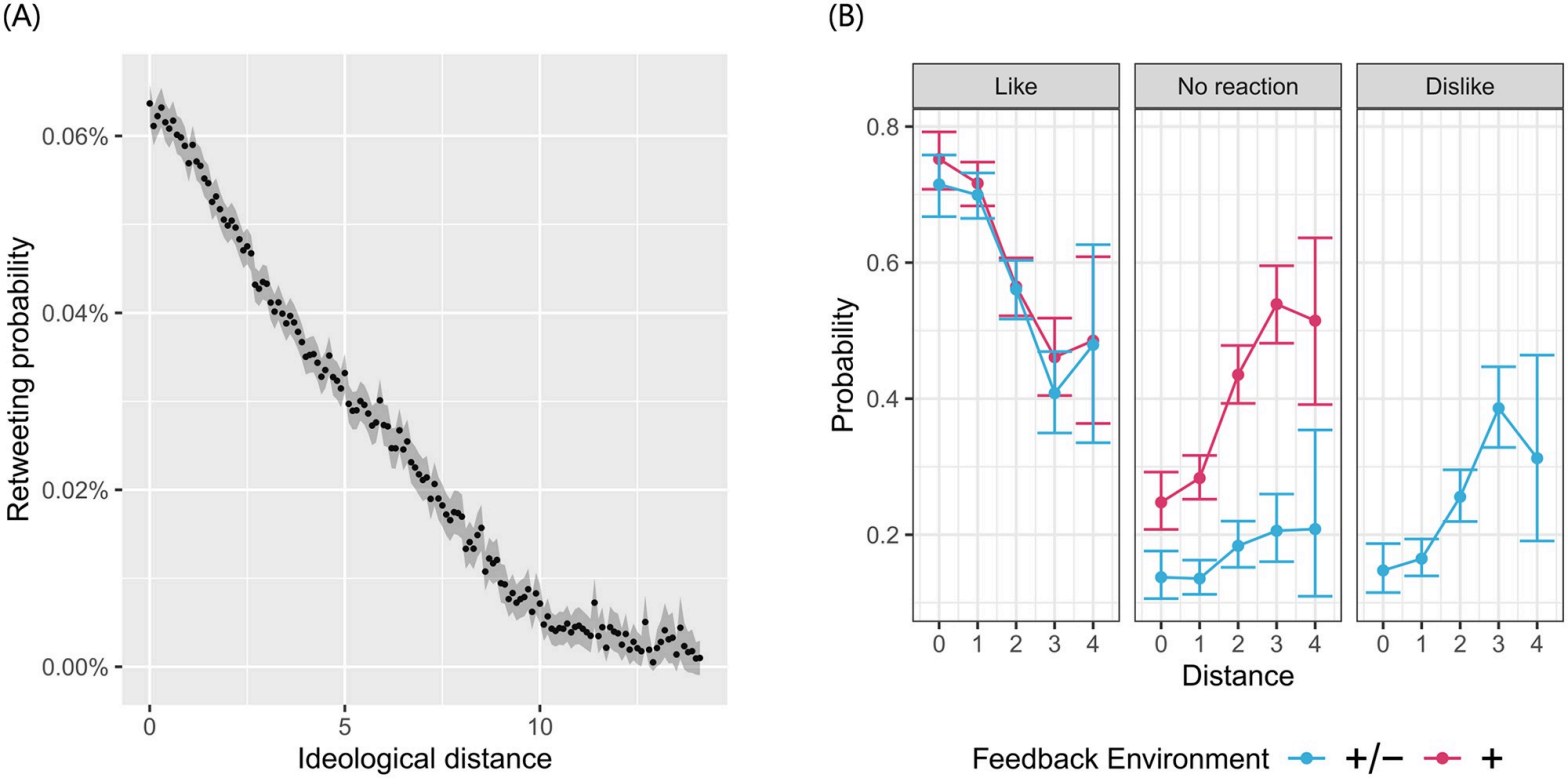
Empirical evidence for the relationship between reactions and ideological distance. **A:** Retweeting probabilities as a function of absolute distance between tweet ideology and the potential retweeter’s ideology. Relative retweeting probability is defined as the tweet’s retweeting probability by a given user, relative to retweeting probability of the same tweet by other users. There are about 120 thousand retweets out of 295 million potential retweeting events (frequency of 0.04%). Each point represents the relative retweeting probability for a 0.1 bin of ideological distance (only bins with at least 100,000 potential retweeting events are depicted). The grey ribbon represents 95% confidence intervals. **B:** Probability of each reaction type as a function of the distance in the two conditions of Experiment 1. *Left panel:* ‘Like’. *Center panel:* ‘No reaction’. *Right panel:* ‘Dislike’. The error bars represent 95% confidence intervals.

Fig 3 confirms our hypothesis by showing that contacts who are ideologically more distant from a given tweet are less likely to retweet it. The graph plots retweeting probabilities as a function of the ideological distance. Each dot represents the average retweeting probability for a 0.1-wide bin of distance. The grey ribbon represents 95% confidence intervals. Estimations of logistic regressions predicting individual retweeting instances show that the results also hold when we control for the baseline propensity of each politician pair to retweet each other (see Section S4 in the S1 File). We do this through a series of fixed effects (see S1 Table in S1 File). In other words, consider a tweet author who writes two tweets with different ideological positions. According to our findings, a contact will be more likely to retweet a tweet that is closer to their ideological position.

### Experiment 1: Reactions by contacts about space travel

The results of the analysis of the Twitter data only concern positive reactions. This is because there is no ‘one-button’ negative reaction on Twitter. Negative reactions only exist through replies. Coding whether a reply consists of a positive or a negative reaction is technically challenging because it requires not only analyzing the text of the reply but also how it relates to the original tweet. These difficulties are compounded by the frequent use of irony. To avoid these potential complications, we measured how the probability of negative reaction changes with the ideological distance between the position of the message and the ideological positioning of the contact in an online study in which participants could provide ‘likes’ and ‘dislikes’.

#### Methods

The online study consisted of three stages: two pre-studies and the main study. In the first pre-study, we asked 100 Prolific participants to provide their opinions about space travel on a one to five-point discrete scale. Specifically, we asked them to indicate the number that best described their position on whether ‘space travel should be allowed’ with one corresponding to ‘Definitely forbidden’ and five to ‘Definitely allowed.’ We then asked them to provide three arguments for their position. At the time of the study—Summer 2021—the topic of space travel received extensive coverage in the media due to several private launches into Earth’s orbit (e.g., Jeff Bezos’s Blue Origin).

In the second pre-study, we showed a different group of Prolific participants (101 participants) the arguments we collected in the first pre-study and asked them to indicate the number, on the same one-to-five scale, that best described the position expressed in the argument. We also asked the participants to judge how elaborate the argument was. Each participant judged 30 arguments and each argument received 10 ratings. We calculated the average value selected for each argument and used the position (out of the five) closest to the average as the position best representing the argument. We then selected the 20 most elaborate arguments for each position, for a total of 100 arguments (based on the average ‘elaborate’ score). This allowed us to obtain the position of each statement on an opinion space ranging from strong opposition (‘Space travel should be definitely forbidden’) to full support (‘Space travel should be definitely allowed’), coded on the 1 to 5 range.

In the main study, we asked 200 Prolific participants to, first, provide their opinions on the topic of space travel and, then, to provide feedback to the arguments. There were two conditions in the study. In the ‘+/−’ condition, participants could provide feedback in the form of likes or dislikes. In the ‘+’ condition, participants could provide feedback only in the form of likes. In both conditions, participants also had the option to provide no feedback. Participants saw the argument on the screen and had a choice between either two (‘+’ condition) and three buttons (‘+/−’condition): a like button denoted by a ‘thumbs up’ emoticon, a dislike button denoted by a ‘thumbs down’ emoticon (this was available only in ‘+/−’ condition), and ‘no feedback’ denoted by an emoticon with a crossed-out circle. Each participant reacted to 20 arguments, and each argument received 20 instances of feedback in each of the conditions (including ‘no feedback’).

At the end of the second pre-study and the main study, we collected demographic information about the participants (age, gender, level of education) as well as habits of social media use. Specifically, we asked participants how often they leave positive comments and negative comments on social media posts. The scale ranged from ‘More than 100 times a day’ to ‘Never.’ Additionally, to measure participants’ attitudes toward a quick low-cost feedback option, we asked participants to indicate whether ‘it would be desirable to have a dislike button’ on a scale from ‘extremely undesirable’ to ‘extremely desirable.’ We also asked participants to explain their responses in a text field.

#### Results

Fig 3 shows the probability of reaction as a function of the distance between the participant’s opinion (provided at the beginning of the experiment) and the position of the argument obtained in the second pre-study. This relationship is negative for positive reactions: participants were more likely to click ‘like’ when a statement was close to their position. The relationship was positive for negative reactions: people were more likely to click ‘dislike’ when a statement expressed a position distant from theirs.

### Experiment 2: Feedback perception by content producers

#### Methods

To validate the linear formulation of the overall feedback (Eq 4), we ran an online study in which we elicited reactions of online participants to different combinations of positive and negative reactions. Ninety-eight Prolific participants completed the study online (the study was run in the Winter of 2022). After providing consent and reading the basic instructions of the experiment, we asked participants to report the maximum, minimum, and average number of likes they get for their posts on social media. The participants were screened based on their social media use as declared to Prolific: only those who were users of Facebook, Twitter, or Instagram were allowed to participate.

First, we asked participants to report their opinion about space travel and to provide a statement in support of their opinion. They were asked to think of the statement as a social media post designed to convince others of their opinions. Then we asked participants to imagine how they would respond to various counts of reactions from other users of the social media platform. Each participant reacted to 36 situations that consisted of different combinations of counts of ‘likes’ and ‘dislikes’. Specifically, after observing reaction counts, participants were asked to indicate on a slider how likely they were to post a similar statement in the future (from ‘Very unlikely’ to ‘Very likely’, coded in the 0 to 100 range).

There were two between-participant conditions. In the ‘+’ condition, participants received only positive feedback in the form of a number of likes. In the ‘+/−’, participants received both a number of ‘likes’ and a number of ‘dislikes.’ Each participant reacted to 36 feedback instances: those in ‘+/−’ reacted to combinations of likes and dislikes; those in ‘+’ reacted only to likes (the corresponding dislikes were not shown to them). These 36 instances were all possible pairs of six levels of ‘likes’ and ‘dislikes.’ In both conditions, the actual numbers of ‘likes’ and ‘dislikes’ were re-scaled in accordance with what the participant reported to be the maximum and the minimum number of likes they received on their posts. The minimum number reported corresponded to 1 on the scale and the maximum number corresponded to 6. The remaining levels were re-scaled so that they equally spanned the range between the minimum and maximum. At the end of the experiment, we administered the same demographic survey as in Experiment 1.

#### Results

First, we discuss the results from the ‘+/−’ condition. To validate our assumption about the form of the net feedback, we regressed the reported likelihood to post a similar statement on the level of likes and the level of dislikes (Model 1 in Table 1). The model has three parameters: the intercept, the weight of likes, and the weight of dislikes.

**Table 1:** Results of Experiment 2. All models have an intercept, participant fixed effects, and errors clustered by participant. 95% confidence intervals are reported in brackets.

	Reported likelihood to post a similar post			
	‘+/−’			‘+’
	1	2	3	4
Like	6.04 [4.65,7.44]			7.88 [6.17,9.58]
Dislike	-5.29 [-6.59,-3.99]			
Difference		5.67 [4.43,6.91]		
$\frac{L}{L+D}$			73.81 [57.6, 90.0]	
Observations:	1728	1728	1728	1800
MSE	286.93	287.76	284.83	176.12
*R* ^2^	0.396	0.394	0.401	0.507

To assess the effects of the two types of feedback on the reported likelihood of posting a similar statement, we regressed the reported likelihood on the difference between the level (out of 6) of likes and the level (out of 6) of dislikes (Model 2) or the ratio of likes in the overall number of feedback instances (Model 3, also using the levels rather than the actual number the participant observed). Both regression models have two main parameters: the intercept and the slope of the difference or the ratio. In all regressions, we included participant-fixed effects to account for individual differences and errors clustered on the level of the participant. Regression results are reported in Table 1.

We found that the fit of the two models with equal weights (Models 2 and 3) was practically the same: *R*^2^s for the linear and ratio models was 0.3948 and 0.401 respectively. These findings are in contrast with existing findings by [32] where the ratio model was found to be superior. It is possible that the failure to replicate these results came from differences in the designs of the studies. Specifically, in order to account for participants’ popularity on social media, we asked the participants to provide the minimum and maximum likes that they have received in the past. Then, we adjusted the number of likes and dislikes they would see to those limits. That implies that the magnitude of the feedback shown to participants had a symmetrical nature. Had we reduced the range of the number of dislikes, we might have found that participants were more sensitive to negative feedback. Because this does not create issues for the main claim of this paper, we leave this for further research.

Second, we performed a similar analysis for the ‘+’ condition. We regressed the reported likelihood of posting a similar statement on the level of likes. We included participant fixed effects and clustered the errors by participant. As expected, the level of likes has a significant positive relationship with the reported likelihood (*b* = 7.88, *p* − *value* < 0.001; for details, see Model 4 in Table 1).

## Experimental test of the main model prediction

In this section, we provide empirical evidence for the main prediction of the model about the extremization of expressed opinions. Specifically, we show that when feedback is asymmetric, a larger proportion of participants choose extreme options than when feedback is symmetric. The task environment closely follows the model setup: participants face a multi-armed bandit task with options that have the same payoff distributions as in the simulations, and select options as a function of the feedback they receive and are not asked to express opinions. In other words, the aim of this study is to provide a conceptual test of the model predictions and show that the dynamic of human *choices* is consistent with the model predictions.

### Experiment 3: Choices in different feedback environments

#### Methods

Participants faced a 5-armed bandit task in which they were instructed to maximize their cumulative payoffs. Six hundred Prolific participants completed the study online (the study was run in the Spring of 2022). After giving consent and reading the general instructions, participants faced repeated choices between five options. The goal of the participant was to maximize their total payoff over 20 rounds. A bonus proportional to the obtained payoff was awarded at the end of the experiment. The participants were assigned one of two conditions: the ‘+/−’ condition and the ‘+’ condition. In the ‘+/−’ condition, payoffs were determined by the numbers of ‘green points’ and ‘red points’ where green points positively affected the bonus and red points negatively affected the bonus. In the ‘+’ condition, the payoff was determined only by the number of green points. The green and red points were generated by our model. Each simulated contact would give a like (+1 green point) or a dislike (+1 red point) with probabilities determined by Eqs 2 and 3. We generated the feedback distributions by assuming there were 50 contacts with opinions uniformly distributed across the five options, with the same set of parameters as in the computer simulations reported on Fig 1A; *d*_0_ = *l*_0_ = 0.9 and *s*_*l*_ = *s*_*d*_ = 1 in the ‘+/−’ condition; *d*_0_ = 0 and *l*_0_ = 0.9, *s*_*l*_ = 1 in ‘+’ condition. After the choice sequence, participants indicated the impact on their bonus from selecting each option (on a scale from “Highly negative” to “Highly Positive”).

#### Results

Panel B on Fig 1 shows the evolution of the choice proportions. For visual clarity, we averaged proportions by blocks of four periods. Consistent with the model predictions, the proportion of extreme choices (options 1 and 5) becomes lower in the ‘+/−’ condition than in the ‘+’ condition. Specifically, only 13% of choices are extreme in the ‘+/−’ condition compared to 27% in the ‘+’ condition. These patterns provide clear evidence for the causal effect of the feedback environment on the proportion of extreme choices.

## Analyses of boundary conditions based on simulations of the learning model

### Interpretation of reactions by contacts

The main result of this paper relies on the assumption that content producers want to avoid negative reactions. The perception of negative reactions from ‘the other side’ might be perceived as positive feedback because it is seen as a confirmation of a person’s beliefs about the (sometimes ‘stupid’ [33]) ‘other side.’ Therefore, the assumption that people want to avoid negative reactions might not apply to those in the extremes—voices that sometimes seem to invite backlash for publicity.

To explore this possibility, we simulated a model where those content producers who express extreme opinions perceive negative reactions as positive rather than negative feedback. This change leads to a reversal of our main result: extreme opinion expressions become more frequent in the ‘+/−’ environment than in the ‘+’ environment (see Section S3.1 in S1 File).

### Aggregation of counts of negative and positive reactions into feedback

We assumed that counts of positive and negative reactions were integrated into overall feedback using a linear model with the same weights for positive and negative counts. A large body of research, however, has documented that people tend to recall negative information better and weigh it more heavily than positive information [34, 35]. Additional simulations show that a higher weight of negative reactions strengthens the effect of feedback on extreme opinion expression.

Recent research suggests that reaction counts might not be aggregated linearly, but using a ratio rule [32]. Simulations with this rule (see Section S1 in S1 File) lead to results very similar to those obtained with the linear model with *δ*_*l*_ = *δ*_*d*_ = 1 (see Section S3.2 in S1 File). The fits of the ratio rule and linear model to the data from Experiment 2 were practically the same (see Table 1).

### Propensity to give negative feedback

The claim that opinion expression is less extreme in the symmetric feedback environment relies on the assumption that contacts would give negative reactions if there was a ‘one-button’ way of doing so. It is probable, however, that people are less willing to provide negative feedback than positive feedback. Most people do not particularly enjoy being negative towards others, especially in public social interactions such as on social media. Therefore, they would be less likely to provide negative reactions than positive reactions. This hypothesis finds support in the survey data we collected as a part of our online studies (see Section S6 in S1 File). Additional simulations show that, unsurprisingly, the moderating effect of introducing the possibility of negative reactions is limited by contacts’ propensity to use it (see Section S3.3 in S1 File).

### Distribution of feedback givers on the opinion space

In our baseline simulations, we assumed that the distribution of contacts’ positions on the ideological spectrum was uniform. We made this assumption for simplicity, but it does not generally hold in naturally occurring environments. A large body of research has shown that in Western societies, political opinion has become more polarized— different parts of the society have very different stances on societal and political issues [36–38]. At the same time, on other issues, the popular opinion converges with a majority supporting the same stance (e.g., the majority of Americans believe that the government should do more regarding climate change [39]).

We analyzed how changing opinion distribution of contacts affects our main result. Additional simulations of our model with an unimodal distribution of contact positions imply that when the popular opinion is largely moderate (i.e., the moderate position is the most popular), adding the possibility of negative reaction has a weaker effect on the proportion of extreme opinion expressions. Intuitively, this is because extreme opinion expressions receive less negative feedback (there are few contacts with extreme opposite opinions). Simulations using a bimodal opinion distribution of contact positions, with the majority of contacts supporting one of the extreme opinions (88%), imply that when popular opinion is polarized, adding the counts of negative reactions has a strong negative effect on the expression of extreme opinion (see Section S3.4 in S1 File).

### Sensitivity of choice to reaction counts

In our simulations, we assumed that the sensitivity of the choice of message position to the reaction counts was the same in the ‘+’ and ‘+/−’ environments. If this does not hold and instead it was lower in the ‘+/−’ environment, this could imply that moving from the ‘+’ environment to the ‘+/−’ environment would not change the proportion of extreme opinions. This is because such a difference in sensitivity could compensate for the difference in how centrally ‘peaked’ the feedback distributions are (Fig 2). Simulations of our model show that *c* has to be 2.5 times larger in the ‘+’ for the two proportions of extreme opinion expression to be the same in the two environments. Analysis of the data collected in Experiment 2 does not show such a drastic difference in sensitivity to feedback between the two environments. A regression analysis estimated the ratio to be 1.4 which is substantially lower than the value of 2.5 necessary for equivalence (see Table 1).

It is worth noting that, from the perspective of the information content of the reaction counts, audience reaction is a more reliable source of feedback in the ‘+/−’ than in the ‘+’ environment. In the ‘+’ environment, an absence of a positive reaction could result from an absence of exposure to the message or contacts not liking it. This, in turn, makes the mapping between how much contacts like the message and the count of ‘+’ reactions noisy. By contrast, in the ‘+/−’ environment, even if the reach is not available, the proportion of ‘+’ reactions is a reliable signal of how much contacts who saw the message liked it.

## Discussion

Our results suggest that making quick low-cost negative reactions available to users of social media and providing counts of such reactions to content producers has the potential to decrease extreme opinion expression. As we discussed in the previous section, this claim is only valid if a set of boundary conditions are satisfied in a particular context. Granted this limitation, our results have potential implications for between-group polarization that we discuss next.

### Implications for between-group polarization

**Fig 4:**
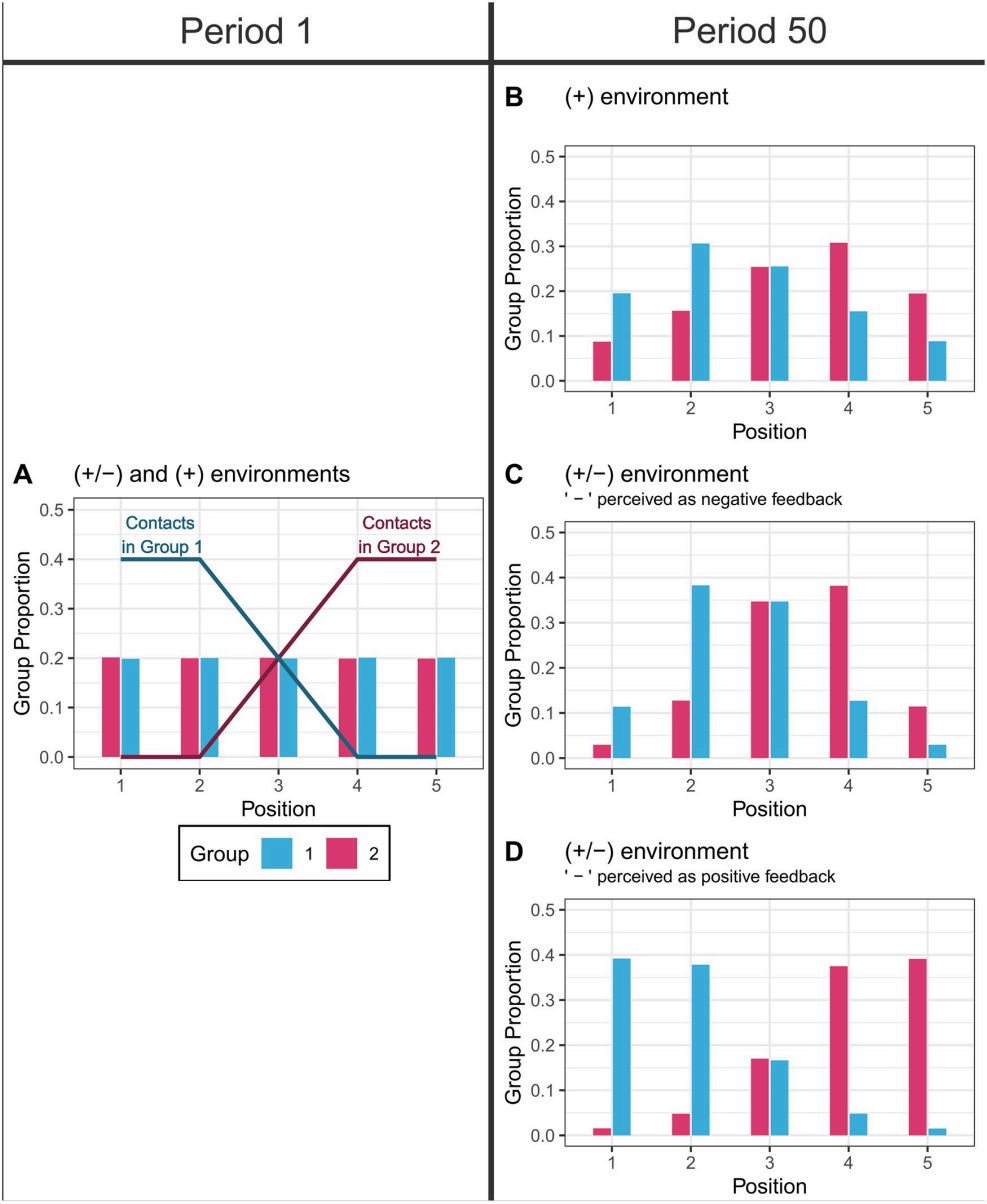
Polarization between two groups with opposing identities. The solid lines represent the distribution of the contacts in the two groups and the vertical bars represent the choice distributions of content producers in the two groups. **A:** Period 1 in the ‘+’ and ‘+/−’ environments. **B:** Period 50, ‘+’ environment. Proportion of extreme opinion expression: 38%. Polarization strength (average pairwise distance between the positions of posted messages): 1.5. **C:** Period 50, ‘+/−’ environment, when negative reactions are perceived as negative feedback. Proportion of extreme opinion expression: 19%. Polarization strength: 1.26. **D:** Period 50, ‘+/−’ environment, when negative reactions are perceived as *positive* feedback. Proportion of extreme opinion expression: 0.54. Polarization strength: 2.27.

Ancillary analyses suggest that providing counts of negative reactions can limit opinion polarization between social groups. To show this, we adapted our model to a setting in which content producers and their contacts belong to two social groups. Content producers are equally likely to belong to either group. Each group contains the same number of contacts, but contacts from the two groups occupy different sides of the ideological spectrum (see Fig 4, panel A, and Section S1 in S1 File). As social media users tend to be more strongly connected to members of their social groups than to members of other groups [1, 40], we assume that contacts from the other group are less likely to react to the messages of the content producers. Simulations show that in both the ‘+’ environment and the ‘+/−’ environment, expressed opinions become polarized. But what is important is that expressed opinions are more moderate and less polarized in the ‘+/−’ environment (Fig 4, panels B and C).

#### Relation to the literature on information exposure

The proposal that the feedback provision mechanism that prevails on popular social media platforms could contribute to polarization relies on a mechanism that differs from mechanisms focused on the nature of the information samples to which people are exposed [41, 42] such as the literature on ‘filter bubbles’ [22, 23]—a common explanation for political polarization that argues that people are mostly exposed to ideas consistent with their opinions and lack exposure to contrarian ideas due to the algorithms behind social media feeds prioritizing like-minded content [43] or people wanting to avoid content with opposing opinions. It is important to note that both mechanisms are not incompatible but potentially complement each other.

Accordingly, it carries different implications about what can be done to limit polarization. Explanations that focus on information exposure imply that social media connections should be altered such that social media users are exposed to contrarian opinions and news. By contrast, the feedback-based mechanism on which we focus suggests that changing the success metrics provided to content producers has the potential to reduce polarization.

#### Relation to the literature on ‘backfire effects’

Recent research has found that exposure to contrarian opinions and information can ‘backfire.’ For example, [24] found that exposure to more liberal content resulted in Republicans taking a more conservative stance. The same, however, was not true for Democrats: they did not shift more to the left after exposure to right-leaning content. This suggests that this effect might not be universal but appears under certain conditions (establishing these requires more research).

In any case, the possibility of a backfire effect induced by exposure to contrarian information raises the possibility that adding a one-button negative feedback option could also ‘backfire’ (especially when the identity of the source of information is known [44]) and lead to more between-group polarization.

This could happen because, when people belong to two groups with oppositional identities, they might enjoy producing content that bothers members of the other group. In this case, they might interpret negative reactions from members of the other group as positive feedback. This situation is similar, to an extent, to the setting we analyzed above in which content producers at the extreme of the opinion spectrum would interpret negative reactions as *positive* feedback. But that setting did not consider group membership. Computer simulations show that, in the ‘+/−’ environment, polarization becomes not only stronger than in the baseline case but also stronger than in the ‘+’ environment (see Fig 4, panel D).

It is important to note that the backfire effect considered here requires that the group identity of those who post reactions is easily visible by content producers and taken into account by them as they respond to feedback. This is because the group identity of the feedback giver is what drives the interpretation of the count of ‘+’ (and ‘−’) reactions.

We believe this assumption is generally not satisfied on most popular social media platforms, because what is most visible are the counts of reactions rather than the identity of those who gave them. And even if some identifying information is available (e.g., it is possible to see the user name and the type of reaction in a few clicks), their group identity might not be easily inferred without additional information about the user. Testing the extent to which users can actually infer the group identity of feedback-givers requires tracking what kind of information users access about others as well as testing how easy it is for them to infer group identity from that information. To the best of our knowledge such information is not available to researchers via the APIs used to download social media data for research. Therefore, we believe that such an investigation along with a direct test of whether a backfire effect affects interpretations of counts of negative reactions should be conducted by the social media platforms via small-scale design change interventions before deciding whether to implement our suggestion to provide a one-button negative feedback option.

### The role of social media platforms

Facebook, Instagram, Twitter, and TikTok neither provide users with a one-button negative reaction nor do they communicate explicitly other instances of negative feedback, such as when someone ‘unfollowed’ a user. At the same time, YouTube has a ‘dislike’ button but only provides the count information to content producers and Reddit makes the counts of ‘downvotes’ available to all.

The hesitancy to include one-button reactions by some of the leading social media platforms likely comes from the assumption that including counts of negative reactions harms user engagement and, therefore, advertising revenues. Most social media platforms have two types of users: those who focus on producing content and those who mostly consume or share content produced by others, but do not (or hardly) produce content. Platforms differ in terms of the proportion of each type. For example, YouTube has a relatively small amount of content producers in comparison to Twitter or Facebook. Both types are important to the functioning of the platform but the effects of including one-button reactions on their engagement on the platform likely differ.

As we saw in Experiment 2, content *producers* aim to avoid negative reactions. Our model assumed that they respond to negative reactions by changing the nature of their posts. But they could also post less content and possibly stop using the platform altogether [9]. Although the negative effect of ‘dislikes’ on the satisfaction derived by users from using social media cannot be eliminated,—it is likely that people would be negatively affected by negative reactions—it is possible to reduce the strength of social influence in the decision to give a ‘dislike’ [45]. This will limit the prevalence of those ‘dislike’ instances that, while negatively affecting user engagement, fail to provide valuable signals to content producers who aim to learn to produce more appealing content. For example, Youtube’s decision to hide the ‘dislike’ count [46] has likely increased the informational value of the ‘dislike’ count as it prevented the snowball effect of users disliking content because others did.

At the same time, content *consumers* might prefer to see the counts of negative reactions as this has informational value for them that pertains to the quality of the content or its relevance. After the change at YouTube was introduced, a lot of users were not happy about it and wanted to reverse it [47]. Users used the negative counts to make a decision whether or not to watch it [48]. On Reddit, users downvote (the Reddit equivalent of a dislike) posts that are either not relevant to a particular community or violate its policies.

This implies that the effect of introducing a one-button negative reaction option on overall user engagement likely differs across platforms. Those that rely on user-produced content might be less susceptible to adopting this design change. Uncovering what can be done to make them more likely to adopt it is an interesting avenue for future research.

## Conclusion

Our study shows that a simple design choice by social media platforms has the potential to affect the tone of online conversations. Providing users with a one-button option to communicate negative reactions could, under the sufficient set of conditions analyzed in this paper, lower the users’ propensity to express extreme opinions.

An important limitation of our analysis based on simulations of a computational model, with respect to normative recommendations, is that it focuses on a single mechanism according to which social media users change their opinions and adapt the nature of the content they post: the quantitative feedback they obtain for their social media posts via counts of positive and negative reactions. We excluded other opinion change mechanisms such as those that entail learning from the content shared by other users, in the form of opinion statements or links to news content, or textual feedback in the form of replies. We excluded other mechanisms in order to develop a clear understanding of the dynamics implied by the feedback-based mechanism. This focus on a single mechanism implies that even if our model assumptions are correct, reliably predicting the effect of providing users with a one-button option to communicate negative reactions on the distribution of expressed opinions, in a particular empirical setting, requires studying how this design change would affect not only the quantitative feedback received by content producers but also how it would affect the other opinion change mechanisms we left out of our analysis. Doing so is an interesting avenue for further research.

This paper contributes to the ongoing discussion about the extent to which social media contributes to political extremization and polarization. We hope our study will point the attention of designers and managers of social media platforms, as well as that of the public, to the potential effects of a seemingly innocuous design feature of social media platforms. It may very well be that providing users with a one-button way of communicating their disagreement with a post could reduce the amount of extreme opinion statements posted on social media. If this is the case, this simple design change might lead to a healthier debate culture on social media.

## Supporting information

S1 File Supplementary material for ‘social media feedback and extreme opinion expression’. (PDF)

## References

[1] Barberá P, Jost JT, Nagler J, Tucker JA, Bonneau R. Tweeting From Left to Right: Is Online Political Communication More Than an Echo Chamber? Psychological Science. 2015;26(10):1531–1542. doi: 10.1177/0956797615594620 26297377

[2] Burrow AL, Rainone N. How many likes did I get?: Purpose moderates links between positive social media feedback and self-esteem. Journal of Experimental Social Psychology. 2017;69:232–236. doi: 10.1016/j.jesp.2016.09.005

[3] Sherman LE, Hernandez LM, Greenfield PM, Dapretto M. What the brain ‘Likes’: neural correlates of providing feedback on social media. Social Cognitive and Affective Neuroscience. 2018;13(7):699–707. doi: 10.1093/scan/nsy051 29982823 PMC6121147

[4] Boyd D, Golder S, Lotan G. Tweet, Tweet, Retweet: Conversational Aspects of Retweeting on Twitter. In: 43rd Hawaii International Conference on System Sciences; 2010. p. 1–10.

[5] Kupavskii A, Ostroumova L, Umnov A, Usachev S, Serdyukov P, Gusev G, et al. Prediction of Retweet Cascade Size over Time. In: Proceedings of the 21st ACM International Conference on Information and Knowledge Management. CIKM’12. Maui, Hawaii, USA: Association for Computing Machinery; 2012. p. 2335––2338.

[6] Schöll N, Gallego A, Mens GL. How Politicians Learn from Citizens’ Feedback: The Case of Gender on Twitter. American Journal of Political Science. 2023; p. 1–18. doi: 10.1111/ajps.12772

[7] Suh B, Hong L, Pirolli P, Chi EH. Want to be retweeted? Large scale analytics on factors impacting retweet in twitter network. In: Proceedings—SocialCom 2010: 2nd IEEE International Conference on Social Computing, PASSAT 2010: 2nd IEEE International Conference on Privacy, Security, Risk and Trust; 2010. p. 177–184.

[8] Eckles D, Kizilcec RF, Bakshy E. Estimating peer effects in networks with peer encouragement designs. Proceedings of the National Academy of Sciences. 2016;113(27):7316–7322. doi: 10.1073/pnas.1511201113 27382145 PMC4941475

[9] Chen G, Chen BC, Agarwal D. Social incentive optimization in online social networks. In: Proceedings of the tenth ACM international conference on web search and data mining. ACM; 2017. p. 547–556.

[10] Thorndike EL. The law of effect. The American Journal of Psychology. 1927;39(1/4):212–222. doi: 10.2307/1415413

[11] Sutton RS, Barto AG. Reinforcement learning: An introduction. Campbridge, MA: MIT press; 2018.

[12] Denrell J. Why most people disapprove of me: experience sampling in impression formation. Psychological review. 2005;112(4):951–978. doi: 10.1037/0033-295X.112.4.951 16262475

[13] Fazio RH, Eiser JR, Shook NJ. Attitude formation through exploration: Valence asymmetries. Journal of personality and social psychology. 2004;87(3):293–311. doi: 10.1037/0022-3514.87.3.293 15382981

[14] Le Mens G, Kareev Y, Avrahami J. The Evaluative Advantage of Novel Alternatives: An Information-Sampling Account. Psychological Science. 2016;27(2):161–168. doi: 10.1177/0956797615615581 26701935

[15] Erev I, Barron G. On adaptation, maximization, and reinforcement learning among cognitive strategies. Psychological review. 2005;112(4):912–931. doi: 10.1037/0033-295X.112.4.912 16262473

[16] Erev I, Roth AE. Maximization, learning, and economic behavior. Proceedings of the National Academy of Sciences. 2014;111(Supplement 3):10818–10825. doi: 10.1073/pnas.1402846111 25024182 PMC4113920

[17] Hull CL. Knowledge and purpose as habit mechanisms. Psychological review. 1930;37(6):511–525. doi: 10.1037/h0072212

[18] Lindström B, Bellander M, Schultner DT, Chang A, Tobler PN, Amodio DM. A computational reward learning account of social media engagement. Nature communications. 2021;12(1):1–10. doi: 10.1038/s41467-020-19607-x PMC7910435 33637702

[19] Garz M, Sood G, Stone DF, Wallace J. What Drives Demand for Media Slant? SSRN. 2018; doi: 10.2139/ssrn.3009791

[20] Banisch S, Olbrich E. Opinion polarization by learning from social feedback. The Journal of Mathematical Sociology. 2019;43(2):76–103. doi: 10.1080/0022250X.2018.1517761

[21] Sarközi M, Jütersonke S, Banisch S, Poppe S, Berger R. The effects of social feedback on private opinions. Empirical evidence from the laboratory. PLOS ONE. 2022;17:e0274903. doi: 10.1371/journal.pone.0274903 36197874 PMC9534395

[22] Sunstein CR. # Republic: Divided democracy in the age of social media. Princeton, NJ: Princeton University Press; 2018.

[23] Pariser E. The filter bubble: What the Internet is hiding from you. London, UK: Penguin Group, The; 2011.

[24] Bail CA, Argyle LP, Brown TW, Bumpus JP, Chen H, Fallin Hunzaker MB, et al. Exposure to opposing views on social media can increase political polarization. Proceedings of the National Academy of Sciences of the United States of America. 2018;115(37):9216–9221. doi: 10.1073/pnas.1804840115 30154168 PMC6140520

[25] Flache A, Mäs M, Feliciani T, Chattoe-Brown E, Deffuant G, Huet S, et al. Models of Social Influence: Towards the Next Frontiers. Journal of Artificial Societies and Social Simulation. 2017;20(4):1–31. doi: 10.18564/jasss.3521

[26] Anderson NH. Averaging versus adding as a stimulus-combination rule in impression formation. Journal of experimental psychology. 1965;70(4):39400400. doi: 10.1037/h0022280 5826027

[27] Karelaia N, Hogarth RM. Determinants of linear judgment: A meta-analysis of lens model studies. Psychological bulletin. 2008;134(3):404–426. doi: 10.1037/0033-2909.134.3.404 18444703

[28] Busemeyer JR, Myung IJ. An adaptive approach to human decision making: Learning theory, decision theory, and human performance. Journal of Experimental Psychology: General. 1992;121(2):177–194. doi: 10.1037/0096-3445.121.2.177

[29] Nassar MR, Wilson RC, Heasly B, Gold JI. An Approximately Bayesian Delta-Rule Model Explains the Dynamics of Belief Updating in a Changing Environment. Journal of Neuroscience. 2010;30(37):12366–12378. doi: 10.1523/JNEUROSCI.0822-10.2010 20844132 PMC2945906

[30] Devlin J, Chang MW, Lee K, Toutanova K. BERT: Pre-training of Deep Bidirectional Transformers for Language Understanding. Arxiv. 2018;.

[31] Le Mens G, Kovács B, Hannan MT, Pros G. Using Machine Learning to Uncover the Semantics of Concepts: How Well Do Typicality Measures Extracted from a BERT Text Classifier Match Human Judgments of Genre Typicality? Sociological Science. 2023;10(3):82–117. doi: 10.15195/v10.a3

[32] Cheng J, Danescu-Niculescu-Mizil C, Leskovec J. How Community Feedback Shapes User Behavior. In: Proceedings of the International AAAI Conference on Web and Social Media. vol. 8. AAAI; 2014. p. 41–50.

[33] Hartman R, Hester N, Gray K. People See Political Opponents as More Stupid Than Evil. Personality and Social Psychology Bulletin. 2023;49:1014–1027. doi: 10.1177/01461672221089451 35481435 PMC10302377

[34] Peeters G, Czapinski J. Positive-Negative Asymmetry in Evaluations: The Distinction Between Affective and Informational Negativity Effects. European Review of Social Psychology. 2011;1(1):33–60. doi: 10.1080/14792779108401856

[35] Unkelbach C, Alves H, Koch A. Negativity bias, positivity bias, and valence asymmetries: Explaining the differential processing of positive and negative information. Advances in Experimental Social Psychology. 2020;62:115–187. doi: 10.1016/bs.aesp.2020.04.005

[36] Boxell L, Gentzkow M, Shapiro JM. Greater Internet use is not associated with faster growth in political polarization among US demographic groups. Proceedings of the National Academy of Sciences. 2017;114(40):10612–10617. doi: 10.1073/pnas.1706588114 PMC5635884 28928150

[37] Heltzel G, Laurin K. Polarization in America: two possible futures. Current Opinion in Behavioral Sciences. 2020;34:179–184. doi: 10.1016/j.cobeha.2020.03.008 32391408 PMC7201237

[38] Fiorina MP, Abrams SJ. Political Polarization in the American Public. Annual Review of Political Science. 2008;11:563–588. doi: 10.1146/annurev.polisci.11.053106.153836

[39] Pew Research Center. Two-Thirds of Americans Think Government Should Do More on Climate. Washington, D.C.; 2020. Available from: https://www.pewresearch.org/science/2020/06/23/two-thirds-of-americans-think-government-should-do-more-on-climate.

[40] Conover MD, Ratkiewicz J, Francisco M, Goncalves B, Menczer F, Flammini A. Political Polarization on Twitter. In: Proceedings of the International AAAI Conference on Web and Social Media. vol. 5. AAAI; 2021. p. 89–96.

[41] Denrell J, Le Mens G. Information Sampling, Belief Synchronization, and Collective Illusions. Management Science. 2017;63(2):528–547. doi: 10.1287/mnsc.2015.2354

[42] Le Mens G, Denrell J. Rational learning and information sampling: On the “naivety” assumption in sampling explanations of judgment biases. Psychological Review. 2011;118(2):379–392. doi: 10.1037/a0023010 21480741

[43] Bakshy E, Messing S, Adamic LA. Exposure to ideologically diverse news and opinion on Facebook. Science. 2015;348(6239):1130–1132. doi: 10.1126/science.aaa1160 25953820

[44] Barber M, Pope JC. Does Party Trump Ideology? Disentangling Party and Ideology in America. American Political Science Review. 2019;113:38–54. doi: 10.1017/S0003055418000795

[45] Lorenz J, Rauhut H, Schweitzer F, Helbing D. How social influence can undermine the wisdom of crowd effect. Proceedings of the National Academy of Sciences. 2011;108(22):9020–9025. doi: 10.1073/pnas.1008636108 21576485 PMC3107299

[46] Team Y. An update to dislikes on YouTube; 2021. Available from: https://blog.youtube/news-and-events/update-to-youtube/.

[47] Brookes T. How to See Dislikes on YouTube Again; 2022. Available from: https://www.howtogeek.com/777886/how-to-see-dislikes-on-youtube-again/.

[48] Boyd K. The Ethical and Epistemic Consequences of Hiding YouTube Dislikes; 2021. Available from: https://www.prindleinstitute.org/2021/12/the-ethical-and-epistemic-consequences-of-hiding-youtube-dislikes/.

