# Supplementary material for: Social media feedback and extreme opinion expression
Source: PLoS One. 2023 Nov 8;18(11):e0293805. doi: 10.1371/journal.pone.0293805 (PMC10631661; doi:10.1371/journal.pone.0293805)
Supplement: S1 File — (PDF) [file pone.0293805.s001.pdf]

Supplementary Material for 'Social Media Feedback and Extreme Opinion Expression'

Elizaveta Konovalova[1], Gaël Le Mens[2,3,4], and Nikolas Schöll[2]

[1]University of Warwick, Coventry, United Kingdom

[2]Universitat Pompeu Fabra, Barcelona, Spain

[3]Barcelona School of Economics, Barcelona, Spain

[4]Barcelona School of Management, Barcelona, Spain

Supplementary Material for 'Social Media Feedback and Extreme Opinion Expression'

None of the studies reported in this article were preregistered. Deidentified data for both experiments along with a codebook and the data-analysis scripts are posted at `https://osf.io/8xhcm/`. The authors did not collect any identifiable information beyond the Prolific ID.

S1 Model Simulations - Additional Details

In all model simulations, the initial valuations of all positions are the same and set at 0. This implies that in the first period, all positions are equally likely to be chosen. This presumes that the content producer does not have intrinsic preferences for a position and will adjust their choices purely based on feedback. The reported simulations' results are based on $10,000$ runs. Unless otherwise stated, we used the following parameters: $J = 50$, $s_l = s_d = 1$, $b = 0.5$, and $c = 0.3$. Parameters s_l, s_d are sensitivity to distance in eq. 2 and 3 respectively; b is the feedback weight in eq. 5; c is the sensitivity to the valuation in eq. 1. We made the following changes to the parameters or the model in the simulations discussed in different sections of the paper.

In all simulations, we set $l_0 = 0.9$. In the results presented in sections 'The Effect of the Feedback Environment on the Distribution of Expressed Opinions' and 'Boundary Conditions', we set d_0 to 0.9 ('+/−'), 0 ('+') and 0.3 (lower baseline propensity for dislikes). In all other simulations, $d_0 = 0.9$. Figure 2 shows the average feedback received by messages at each position in '+' and '+/−' environments. See Section S4 for simulation results.

In all simulations except those for the discussion of the opinion distribution of the contacts, the opinion distribution of the contacts is uniform (10 for each of the five options). The unimodal distribution is implemented as follows: 20 contacts in position three, 10 in positions two and four, and five in positions one and five. The bimodal distribution is

implemented as follows: 22 contacts in positions one and five, and two contacts in positions two, three, and four.

In all simulations except those about the aggregation of negative and positive feedback, we set $\delta_l = \delta_d = 0.5$. For that section, we simulated the model with $\delta_l = \delta_d = 1$. Moreover, we simulated a model where we defined the overall feedback as a ratio rather than a linear combination of positive and negative reactions:

$$F_{k,t} = \frac{L_{k,t}}{L_{k,t} + D_{k,t}}. \tag{1}$$

To compare the results of the two models, when running simulations, we divided the feedback $F_{k,t}$ by its standard deviation. The standard deviation is calculated using the simulated feedback from 50 contacts to content producers in the case where both were uniformly distributed across the five options. This ensured that the impact on the value of the option was comparable under the two different rules.

To explore the possibility that contacts with extreme opinions seek disagreement, we simulated a version of the model where content producers who express an extreme opinion (one or five) give *positive* rather than negative weight to the number of '−':

$$F_{k,t} = \delta_l * L_{k,t} + \delta_d D_{k,t}, \tag{2}$$

where $\delta_l = \delta_d = 0.5$ are weights of positive and negative reactions. This applies only to the extreme positions; if the content producer chose any other option, the feedback function is that used in the baseline model (eq. 4).

Between-group polarization

To analyse the impact of counts of negative reactions on opinion polarization, we simulated a version of the model where contacts and the content producer belong to one of two groups. Initially, the content producers of the two groups value all 5 positions equally, and their first messages are thus uniformly distributed on the ideological spectrum. The group membership of contacts depends on the side of the ideological spectrum to which the

contact belongs. All contacts in positions (1) and (2) and half of the contact in position (3) belong to one group. All other contacts belong to the other group. In each simulation run, we randomly determined the group of the focal content producer. Moreover, we assume that contacts from the other group have lower baseline propensities to react positively or negatively to the messages of the content producer (contact from the same group as content producer: $l_0 = d_0 = .9$; from the other group $l_0 = d_0 = 0.5$).

To assess the strength of polarization at the end of period t we calculated the average pairwise distance between the positions of the messages posted at time t of the members of different groups [1]. Specifically, we generated 10,000 message positions from each group according to the distribution of positions predicted by the model (see graphs in Fig. 4) and calculated the pairwise distance between these two samples. This metric reflects the average distance on the ideological spectrum between two randomly chosen group members at time t. The lower the average pairwise distance, the closer the two members are to each other.

S2 Analysis of Twitter data - Additional Methodological Details

Retweeting someone else's tweet is a basic form of positive reaction on Twitter. In this analysis, we measure the association between the probability that a contact retweets a tweet and the ideological distance between a tweet and a contact.

We collected the tweets published by all politicians who served in the national parliament of Spain or any of its regional parliaments between the start and the end of the national legislature (from July 2016 to April 2019) and then restricted the sample to the periods during which they were in office (n=1,282 politicians). Our sample of tweets includes original tweets as well as retweets.

We position tweets on the political spectrum space by using a machine learning classifier to compute their typicalities in the 'Right' and the 'Left' (see [4] for an introduction to this method). We took a random half of the politicians and classified their tweets as left-leaning

('L') or right-leaning ('R') based on party membership (501,253 tweets by 643 politicians).[1] This served as a training set to train a deep learning classifier based on the 'BERT' language model [2]. The tweets of the other half of the politicians make the 'prediction set' (481,472 tweets by 639 politicians). The analysis reported in the body of the paper is based on tweets in the prediction set.

The BERT classifier we used is a state-of-the-art model that consists of an artificial neural network with many layers (a 'deep neural network') that takes the text of a tweet as an input and outputs the probability that a tweet has been published by an author who is from a right-wing party ($P(R \mid \text{txt}_m)$, where txt_m is the text of tweet m) and the probability the author is from a left-wing party ($P(L \mid \text{txt}_m) = 1 - P(R \mid \text{txt}_m)$). We used the tweets of a random half of politicians, stratified by L or R political orientation, to train the model. Next, we applied the trained model to the tweets in the prediction set to compute the ideological position of all tweets in this set. We see a 'right-wing' tweet as a tweet with text that strongly suggests it has been written by a right-wing politician. Accordingly, we define the ideological position of tweets (their 'rightwingness') in terms of the log odds ratio that the tweet has been published by a right-wing politician:

$$\mathcal{I}_m = \log \frac{P(R \mid \text{txt}_m)}{P(L \mid \text{txt}_m)} - \log \frac{P(R)}{P(L)}, \tag{3}$$

where $P(R)$ and $P(L)$ are the proportions of tweets published by right-wing and left-wing politicians in the data, respectively. These quantities are scaling factors that do not affect the relative positions of the tweets. Formally, $\mathcal{I}_m$ can also be seen as the difference between the 'BERT-typicalities' in the 'Right' and 'Left' concepts (see [3] for an introduction to this typicality measure and evidence for its empirical validity).

We estimated the ideological positions of politicians ($\mathcal{I}_p$), simply by taking the average position of their tweets:

$$\mathcal{I}_p = \frac{1}{n_p} \sum_{m \in |T|_p} \mathcal{I}_m, \tag{4}$$

[1] See [5] for the classification of parties as Left or Right.

where $|T|_p$ is the set of tweets written by politician p. Because we consider potential retweet events of tweets authored by politicians by other politicians, this approach yields an ideological measure of each tweet and each contact. We define the ideological 'distance' as the absolute distance between the positions of tweet m and contact j:

$$\mathcal{D}_{mj} = |\mathcal{I}_m - \mathcal{I}_j|. \tag{5}$$

The set of potential retweet events is the combination of all tweets and contacts (politicians in the data who are not the tweet author). For these, we have information on the identity of the content producer (writer of the tweet, w), the contact (j), the ideological distance between tweet m and contact j and whether a retweet event actually took place in the 30 days following the publication of tweet m ($Retweet_{wmj} = 1$) or not ($Retweet_{wmj} = 0$). We use the fixed 30-day window and only consider tweets published at least 30 days before the end of our observation period, to avoid censoring issues that would complicate the analyses. This creates a sample of about 295 million potential retweeting events that concern 467,092 tweets published until March 1st, 2019.

We estimate the following logistic regression:

$$Retweet_{wmj} = Logit(\beta * \mathcal{D}_{mj} + \pi_{wj} + \mu_m + \epsilon_{wmj}), \tag{6}$$

where π_{wj} is a writer-contact fixed effect and μ_m is a tweet fixed effect. Results of the regression analysis are in Table S1.

S3 Model Simulations - Additional Results

S3.1 Interpretation of Reactions by Contacts

We simulated a model where those content producers that express extreme opinions perceive negative reactions as positive rather than negative feedback. In the '+/−' environment, the proportion of extreme opinion expression ((1) and (5)) increased from 40% to 81% after 20 periods and the proportion of moderate opinion expression (3) decreased from 20% to 9%.

S3.2 Aggregation of Counts of Negative and Positive Reactions into Feedback

Weight of the negative reaction. In simulations of a version of our model with a larger weight for negative reactions ($\delta_l = 0.5; \delta_d = 1.5$), the final proportion of extreme opinion expression was lower than when positive and negative reactions were equally weighted (5 % versus 10%).

Ratio vs. Linear Rule in Feedback Integration. The simulations of the ratio model show that the proportion of extreme choices decreases from 40% to 30% in period 20. This is comparable to what happens with the baseline model with $\delta_l = \delta_d = 1$ (using re-scaled feedback produced by a linear rule). Simulations show a reduction from 40% to 29% in period 20.

S3.3 Propensity to Give Negative Feedback

In terms of our model, we can capture the asymmetry for the propensity to react negatively by assuming a lower baseline propensity for negative reactions (d_0) than for positive reactions (l_0), in the '+/−' environment. Simulations with $d_0 = 0.3$ and $l_0 = 0.9$ show that, in period 20, the proportion of extreme choices decreases from 40% to 17%. This decrease is not as strong as what was obtained in the baseline simulation of the '+/−' environment (decrease to 10%), but stronger than in the baseline simulation of the '+' environment (decrease to 29%). Unsurprisingly, the moderating effect of introducing the possibility of negative reactions is limited by contacts' propensity to use it.

S3.4 Distribution of Feedback Givers on the Opinion Space

First, we simulated our model using the same parameters as the baseline but with a unimodal distribution of contact positions instead of the uniform distribution assumed in the baseline. The simulations show that the proportion of extreme opinion expression decreased to 14% and 5% in period 20, in the '+' and '+/−' environments, respectively. The between-environment difference is thus smaller (9%) than in the baseline simulations

(19%).

Second, we simulated our model with a bimodal opinion distribution of contact positions, with the majority of contacts supporting one of the extreme opinions (88%). In the '+' environment, the final proportion of extreme opinion expression *increased* to 59%. In the '+/−' environment, this proportion *decreased* to 29%.

S4 Ideological distance and retweeting probability on Twitter - Additional Results

Table S1 reports three different specifications of the logistic regression described in the methods section above (eq. 6). Column (1) reports a model that predicts retweet instances based on the ideological distance between the tweet and the contact and a tweet fixed effect (μ_i). It shows that an increase in ideological distance decreases the retweet probability. Next, we add additional fixed effects to test the robustness of our results. In column (2) we add a contact fixed effect (π_c) to control for the baseline probability of each politician to retweet tweets by others. Finally, in column (3), we add a writer-contact fixed effect (π_{wc}) which controls for the baseline probability of each politician to retweet a given other politician. Note that this strongly reduces the sample size, as we now only rely on variation in ideological distances and retweeting probabilities *within politician pairs*, all observations nested in fixed effect groups without variation in the dependent or independent variable are dropped. Effect sizes are attenuated but always remain strongly significant. This shows that ideological distance is associated with a less positive reaction, even when controlling for the personal relationship between the content producer (tweet author) and the contact and when controlling for tweet characteristics.

S5 Experiment 3: Choices in Different Feedback Conditions - Additional Results

To assess whether the participants acquired the payoff structures implied by the feedback (see Figure 2, we asked them to indicate the impact on their bonus payment from the

selection of each option. Figure S1 shows the boxplots of normalized within participant judgments of impact. The participants' judgments reflect the underlying payoff structure and show that the moderate option (3) is judged as relatively more attractive than the extreme options when negative feedback is available ('+/−' condition) as compared to when negative feedback is not available ('+' condition).

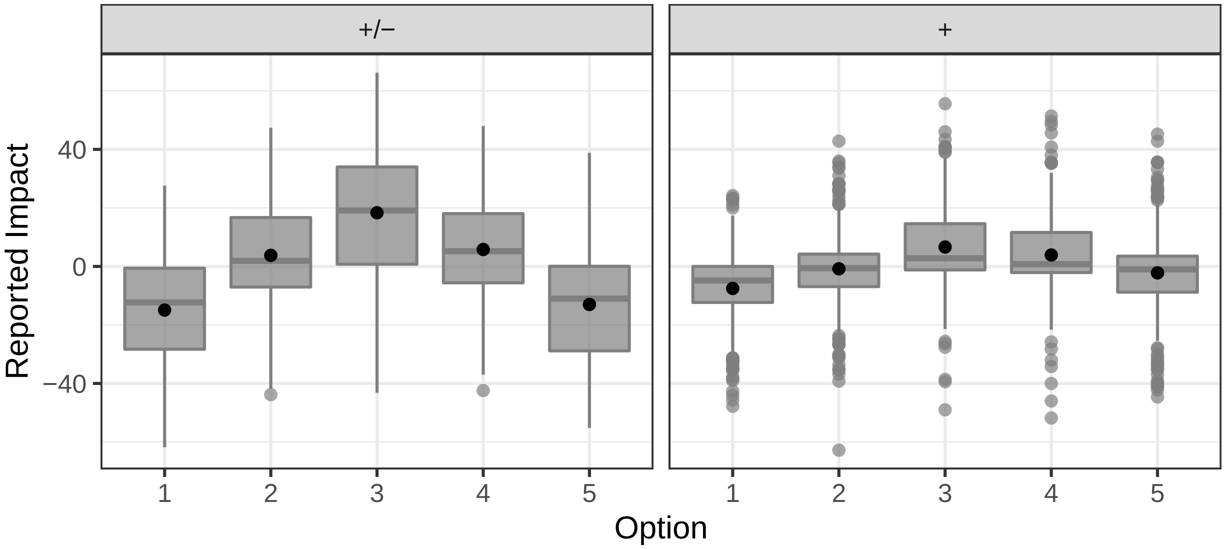

Figure S1

Within-participant normalized judgement of impact selecting of each option on their overall payoff.

S6 Survey about social media use habits

We collected responses to the survey across several experiments (see Methods). Specifically, we collected 399 responses overall in Experiments 1 and 2. Figure S2 shows the distribution of the responses collected across the experiments, specifically, the frequency of posting positive and negative comments as well as the desirability of a dislike button. We asked participants about the frequency with which they write positive or negative comments on social media platforms: 97% of the participants reported leaving negative comments very rarely (every few weeks or less often) compared to only 55% reporting the same thing about

positive comments. We also asked participants whether they thought that a ‘dislike button would be desirable’ and why. The results showed a mild tendency against the ‘dislike button’: 42% of participants responded they found the ‘dislike’ button to be undesirable in comparison to 22% who found it desirable ($N = 399$). Those who were in favour of a dislike button referred to the ability to express disagreement, the opportunity to start a debate, and an increased variety of possible tools for negative feedback as reasons to support a dislike button. Those who were against the dislike button noted that the internet already can be a very negative place, and adding a way to express negative feelings can encourage bullying and lead to detrimental consequences on the mental health of social media users.

Dependent Variable:	Retweet=1		
Model:	(1)	(2)	(3)
Variables			
Ideological distance	-0.3978***	-0.3616***	-0.0415***
	(0.0023)	(0.0020)	(0.0037)
Fixed-effects			
Tweet FE	Yes	Yes	Yes
Contact FE		Yes	
Writer-contact FE			Yes
Fit statistics			
Observations	36,912,846	35,628,072	2,818,615
Squared Correlation	0.00918	0.03977	0.26182
Pseudo R^2	0.09612	0.21470	0.36592
BIC	2,474,127.1	2,284,920.8	1,641,229.1

Clustered (cluster) standard-errors in parentheses

*Signif. Codes: ***: 0.01, **: 0.05, *: 0.1*

Table S1

*Logistic regression of retweet probability on the ideological distance between a tweet and contact. Clustered standard errors are in parentheses. The numbers of observations for each model are lower than the total number of potential retweeting events because sets of observations with no within FE variability in the dependent variable are dropped. Signif. Codes: ***:* $p < 0.001$

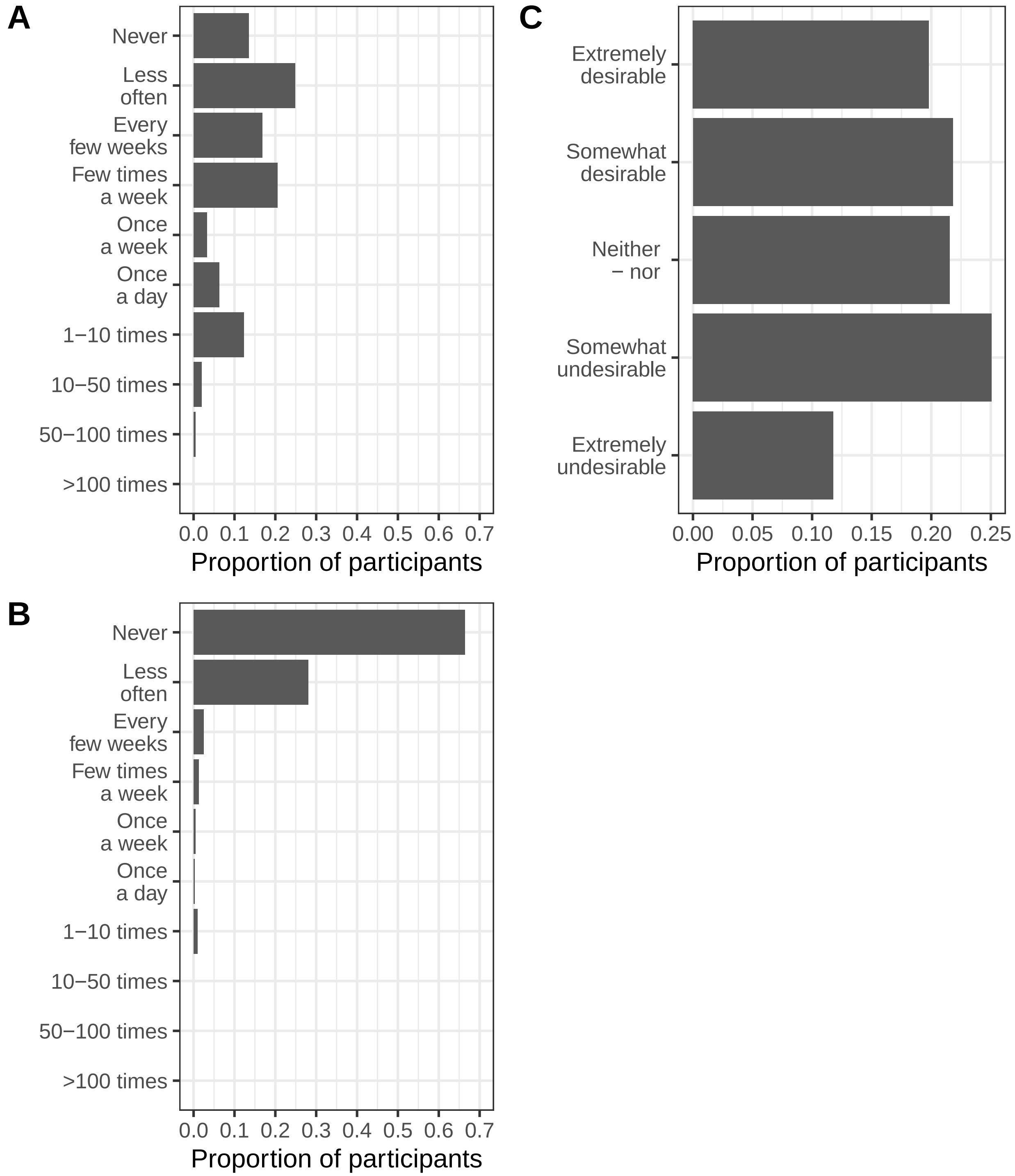

Figure S2

Results of the social media use survey. ***A.*** *Distribution of responses about the frequency of posting positive comments.* ***B.*** *Distribution of responses about the frequency of posting negative comments.* ***C.*** *Distribution of responses about the desirability of a dislike button.*

References

[1] Joshua Becker, Ethan Porter, and Damon Centola. The wisdom of partisan crowds. *Proceedings of the National Academy of Sciences of the United States of America*, 166(22):10717–10722, 2019.

[2] Jacob Devlin, Ming-Wei Chang, Kenton Lee, and Kristina Toutanova. BERT: Pre-training of Deep Bidirectional Transformers for Language Understanding. 2018.

[3] Gaël Le Mens, Balázs Kovács, Michael T. Hannan, and Guillem Pros. Measuring the typicality of text documents using a bert classifier. *Working Paper*, 2022.

[4] Gaël Le Mens, Balázs Kovács, Michael T. Hannan, and Guillem Pros. Using machine learning to uncover the semantics of concepts: How well do typicality measures extracted from a bert text classifier match human judgments of genre typicality? *Sociological Science*, 10(3):82–117, 2023.

[5] Nikolas Schöll, Aina Gallego, and Gaël Le Mens. Politician-citizen interactions and dynamic representation: Evidence from twitter. *Barcelona School of Economics Working Paper no. 1238*, 2021.
